# Marine-Derived Padina Minor Extract Improves Lipid and Glucose Metabolism in Obese Rats: Evidence for PPARγ and ADIPOR1 Modulation

**DOI:** 10.3390/nu18101572

**Published:** 2026-05-15

**Authors:** Anton Bahtiar, Dinda Puspita Dewi Wahyudi, Riani Widiarti, Sirithon Siriamornpun

**Affiliations:** 1 Department of Pharmacology and Toxicology, Faculty of Pharmacy, Universitas Indonesia, Gedung Fakultas Farmasi, UI Kampus, Depok 16424, Indonesia; 2 Department of Biology, Faculty of Mathematics and Natural Sciences, Universitas Indonesia, Gedung Department of Biology Building, UI Campus, Depok 16424, Indonesia; rianiwid@sci.ui.ac.id; 3 Research Unit of Thai Food Innovation, Department of Food Technology and Nutrition, Faculty of Technology, Mahasarakham University, Maha Sarakham 44150, Thailand; sirithon.s@msu.ac.th

**Keywords:** *Padina minor*, PPARγ, ADIPOR1, flavonoids, obesity, nutraceutical

## Abstract

Background/Objectives: Obesity is a multifactorial metabolic disorder characterized by excessive adipose accumulation and dysregulated lipid and glucose homeostasis. Marine brown algae contain diverse bioactive compounds with potential metabolic benefits; however, the in vivo anti-obesity effects of *Padina minor* remain insufficiently characterized. Methods: This study evaluated the effects of *P. minor* ethanolic extract on adipose metabolism and metabolic parameters in obese rats induced by a high-fat diet (HFD). Male Wistar rats (*n* = 36) were rendered obese via HFD and treated with *P. minor* extract (25, 50, or 100 mg/kg BW) for 4 weeks, with orlistat (30 mg/kg BW) serving as a reference control. Body weight, food intake, Lee index, visceral fat mass, serum lipid profile, and glucose levels were assessed, alongside protein expression of PPARγ, CNR1, and ESR1 (ELISA) and gene expression of *Pparγ* and *Adipor1* (qPCR). Phytochemical constituents were analyzed using GC–MS and LC–MS/MS. Results: *P. minor* extract significantly attenuated body weight gain, adiposity indices, and visceral fat accumulation compared with HFD controls (*p* < 0.05), and improved metabolic profiles by reducing total cholesterol, triglycerides, and glucose levels while increasing HDL-cholesterol. At the molecular level, treatment was associated with decreased PPARγ and CNR1 expression and increased *Adipor1* and ESR1 expression. The highest dose (100 mg/kg BW) produced effects comparable to orlistat. Phytochemical analysis identified flavonoids and phenolic acids, including quercetin, catechin, chlorogenic acid, and p-coumaric acid. Conclusions: *Padina minor* ethanolic extract improves metabolic parameters and adipose tissue characteristics in HFD-induced obese rats, potentially through modulation of pathways related to adipogenesis and lipid metabolism, supporting its potential as a marine-derived nutraceutical candidate for obesity management; however, further studies are required to confirm its mechanisms and clinical relevance.

## 1. Introduction

Obesity is a complex metabolic disorder characterized by excessive fat accumulation and dysregulation of lipid and glucose homeostasis, contributing to a wide range of chronic diseases. Beyond a simple imbalance between energy intake and expenditure, obesity is now recognized as a multifactorial condition involving intricate interactions among hormonal, genetic, and molecular signaling pathways [1,2]. The global rise in obesity prevalence highlights the urgent need for effective and safer therapeutic strategies, particularly those derived from natural sources, as current pharmacological treatments are often associated with adverse effects [3,4,5].

Marine organisms, especially brown algae, have emerged as promising sources of bioactive compounds with diverse pharmacological properties. Species within the genus *Padina* are known to contain high levels of polyphenols and flavonoids, which contribute to their antioxidant, anti-inflammatory, and metabolic regulatory activities [6,7,8]. Although *Padina minor* has demonstrated antioxidant potential, its anti-obesity effects and the underlying molecular mechanisms remain poorly characterized, particularly in vivo models. In particular, the roles of key metabolic regulators such as peroxisome proliferator-activated receptor gamma (PPARγ), adiponectin receptor 1 (ADIPOR1), cannabinoid receptor 1 (CNR1), and estrogen receptor 1 (ESR1) in mediating its effects have not been systematically investigated [9,10].

Bioactive compounds commonly found in brown algae, including flavonoids such as quercetin, catechin, and chlorogenic acid, have been reported to influence metabolic pathways related to adipogenesis and lipid metabolism. These compounds can modulate key regulators such as PPARγ and ADIPOR1, thereby inhibiting adipocyte differentiation and promoting lipid oxidation [11]. The combined action of these phytochemicals may exert synergistic effects on multiple molecular targets, contributing to improved metabolic homeostasis [12,13].

However, despite these promising indications, there is a lack of comprehensive in vivo evidence linking *P. minor* phytochemicals to specific molecular pathways involved in obesity-related metabolic dysfunction. Therefore, this study aimed to evaluate the effects of *P. minor* ethanolic extract in a high-fat diet-induced obese rat model and to examine its association with key molecular regulators of adipose metabolism. Specifically, we assessed the expression of PPARγ, ADIPOR1, CNR1, and ESR1 to explore potential pathways underlying its metabolic effects.

To our knowledge, this is the first study to evaluate the in vivo anti-obesity effects of *Padina minor* ethanolic extract in a high-fat diet-induced obesity model, integrating metabolic, biochemical, and molecular analyses. This study further provides novel evidence on the modulation of key regulators of adipose metabolism, including PPARγ, ADIPOR1, CNR1, and ESR1.

## 2. Materials and Methods

Body weight was measured using an analytical balance. Serum biochemical parameters were analyzed using a clinical chemistry analyzer. RNA purity and concentration were assessed spectrophotometrically. All instruments and reagents are listed in Supplementary Table S1.

### 2.1. Preparation of P. minor Extract

#### 2.1.1. Sampling of *P. minor*

Wild *Padina minor* thalli were collected from the intertidal zone of Thousand Island, Jakarta, Indonesia (−5.745426° N/S, 106.616800° E/W) during low tide in January-2024; wet season. Healthy, epiphyte-free fronds were hand-picked at 0–1 m depth and placed in sterile polyethylene bags, rinsed on-site with ambient seawater to remove sand and debris, and transported on ice to the laboratory within 4 h. Species identity was confirmed morphologically by an experienced phycologist using standard taxonomic keys for brown algae, based on diagnostic features including thallus morphology, fan-shaped frond structure, concentric banding, degree of calcification, and surface texture characteristic of the genus *Padina*. Identification followed established taxonomic descriptions for *Padina minor* [14,15]. The identification was further cross-checked against the Algae Base database to ensure nomenclatural accuracy and current taxonomic status. In addition, a voucher specimen (Voucher No. 143810) was deposited at the Oceanography Laboratories, National Research and Innovation Agency (BRIN), Indonesia, and independently verified by taxonomic experts, ensuring traceability and reproducibility of species identification.

In the laboratory, samples were rinsed with distilled water, blotted dry, and shade-dried at ≤40 °C, then milled to a fine powder (≈40-mesh). The powder was stored in amber, airtight containers at −20 °C with desiccant until extraction (≤30 days). Moisture content was measured on a subsample (AOAC 934.01) to report dry-weight–based yields. The extract was stored at −20 °C in airtight, light-protected containers and aliquoted to minimize repeated freeze–thaw cycles prior to use.

#### 2.1.2. Examination of the Chemical Content of *P. minor* Using GCMS

The 70% ethanolic extract of *Padina minor* (50 mg) was reconstituted in 1 mL of n-hexane and filtered through a 0.22 µm PTFE membrane before GC–MS analysis. Separation was performed on a capillary column (30 m × 0.25 mm, 0.25 µm film thickness, 5% phenyl-methylpolysiloxane) using helium as carrier gas (1.0 mL/min). The oven temperature was programmed from 60 °C (1 min hold) to 300 °C at 10 °C/min and maintained for 10 min. Injector and transfer line temperatures were set at 250 °C and 280 °C, respectively, and electron-impact ionization (70 eV) was used with a scan range of 40–600 *m*/*z* [16].

Compounds were identified based on comparison of their mass spectra with the NIST library (match ≥80%) and verification of retention indices (RI) calculated using a homologous series of n-alkanes (C8–C40). Semi-quantitative results were expressed as relative percentage of total ion current (TIC) peak area. To ensure reproducibility, an internal standard (n-eicosane, 10 µg/mL) was added before injection, and blank runs were analyzed between samples to avoid carryover. Major compounds were grouped by class (fatty acids, sterols, phenolics, hydrocarbons), and detailed GC–MS parameters and compound lists are provided in Supplementary Tables S1 and S2.

#### 2.1.3. Examination of the Chemical Content of *P. minor* Using LCMS

The 70% ethanolic extract of *Padina minor* (10 mg/mL) was filtered through a 0.22 µm PTFE syringe filter and analyzed using liquid chromatography–mass spectrometry (LC–MS, UHPLC Thermo Scientific model Accela-Markham, ON, Canada). Separation was achieved on a reversed-phase C18 column (100 × 2.1 mm, 1.7 µm) maintained at 40 °C. The mobile phase consisted of (A) 0.1% formic acid in water and (B) 0.1% formic acid in acetonitrile, with a gradient of 5–95% B over 30 min at a flow rate of 0.3 mL/min. The injection volume was 5 µL [16].

Mass spectrometric detection was performed using an electrospray ionization (ESI) source operated in both positive and negative ion modes under the following conditions: capillary voltage 3.5 kV, desolvation temperature 350 °C, source temperature 120 °C, and scan range *m*/*z* 100–1500. Nitrogen was used as both nebulizing and desolvation gas.

Data acquisition and processing were performed using Xcalibur software 4.7. Tentative compound identification was achieved by comparing accurate masses, isotopic patterns, and MS/MS fragmentation spectra with data from public databases MassBank and published literature. A match tolerance of ±5 ppm for precursor ions and a fragment match ≥80% was applied.

Compounds were grouped by chemical class, and their relative abundances were expressed as normalized peak intensities (% of TIC). A detailed list of tentatively identified compounds, including molecular formulae, retention times, and mass spectral data, is provided in Supplementary Table S3.

### 2.2. Animal Treatment

#### 2.2.1. Randomization and Blinding

Following ARRIVE 2.0 guidelines [17], rats were the experimental unit. After acclimation, baseline body weights were recorded, and animals were stratified into tertiles (light/medium/heavy). Rats were obtained from a certified laboratory animal breeder and were healthy, non-genetically modified animals with no prior experimental procedures. Animals were maintained under standard laboratory conditions prior to and during the study. Within each tertile, rats were allocated to six groups (Normal, HFD, HFD + Orlistat 30 mg/kg BW, *P. minor* 25/50/100 mg/kg BW) using computer-generated permuted blocks of 6 to ensure balance across groups. Treatments were prepared as coded suspensions (A–F) in 0.5% CMC-Na with identical volume and appearance; dosing syringes were opaque. Outcome assessors were blinded to group allocation for all measurements (body weight, Lee/adiposity indices, serum lipids and glucose, ELISA for PPARγ/CNR1/ESR1, qPCR for ADIPOR1/PPARγ, and histology). Sample tubes, plates, and slides were labeled with codes only, and plate well positions were randomized to minimize batch/edge effects. Unblinding occurred only after the primary statistical analysis plan was executed and figures finalized.

#### 2.2.2. Ethical Statement

All animal procedures were conducted in accordance with the ARRIVE 2.0 guidelines and approved by the Ethics Committee of the Faculty of Medicine, Universitas Indonesia–Cipto Mangunkusumo Hospital (approval No. KET.267/UN2.F1/ETIK/PPM.00.02/2024). The study adhered to the principles outlined in the Guide for the Care and Use of Laboratory Animals [18] and the institutional animal welfare regulations. Thirty-six healthy male Wistar rats (4 weeks old, 100–120 g) were obtained from a certified breeder and acclimatized for one week before the experiment. Animals were housed under controlled environmental conditions (22 ± 2 °C; 12 h light/dark cycle; 55 ± 10% humidity) with ad libitum access to water and standard chow. Animals were housed under identical conditions, and cage positions were standardized to minimize environmental variability. Randomization and blinding procedures were applied as described in Section 2.2.1 to minimize bias in group allocation and outcome assessment. All efforts were made to reduce the number of animals used and to minimize discomfort throughout the study.

#### 2.2.3. Animal Grouping

After one week of acclimatization, thirty-six male Wistar rats (4 weeks old, 100–120 g) were randomly assigned to six groups (*n* = 6 per group). Obesity was induced by administration of a high-fat diet (HFD) for 90 days. During this period, HFD-fed rats developed obesity characterized by approximately 50% body-weight gain and increased Lee index values compared with the normal control group. Although several animals reached the obesity criterion before day 90, all HFD-fed groups underwent the same 90-day induction period to ensure consistent metabolic conditioning prior to treatment initiation. Animals then received the following interventions for four weeks: NC = the group that was given standard feed (non-HFD) of 58 kcal/20 g/rat, HFD = the group that was given HFD feed of 89.72 kcal/20 g/rat; ORL = the group that was given Orlistat 30 mg/kg BW; PM-L the group that was given *P. minor* extract 25 mg/kg BW; PM-M = the group that was given *P. minor* extract 50 mg/kg BW, PM-H = the group that was given *P. minor* extract 100 mg/kg BW.

All treatments were administered orally once daily in 0.5% CMC-Na (vehicle). Orlistat served as the reference anti-obesity drug. Body weight, food intake, and Lee index were recorded throughout the study.

### 2.3. Examination of Body Weight, Food Intake, and Lee Index

Body weight and food intake were recorded weekly throughout the experimental period. The amount of food consumed was calculated as the difference between the feed provided and the remaining feed after 24 h, averaged per rat per day [16]. A higher Lee index indicates greater adiposity. All measurements were performed by blinded assessors to minimize observer bias.

### 2.4. Serum Biochemical Analysis

At the end of the treatment period (day 91), rats were anesthetized with ketamine (70 mg/kg BW, i.p.) and xylazine (8 mg/kg BW, i.p.). Blood samples were collected from the retro-orbital sinus using heparinized capillary tubes. Serum was obtained by centrifugation at 2500 rpm for 10 min at 4 °C and stored at −20 °C until analysis.

Serum concentrations of triglycerides (TG), total cholesterol (TC), high-density lipoprotein (HDL), and glucose were measured using commercial enzymatic colorimetric kits according to the manufacturer’s instructions.

All biochemical assays were performed in duplicate by blinded assessors using calibrated instruments (see Supplementary Table S1 for equipment and reagent details).

### 2.5. Examination of PPARγ, CNR1, and ESR1 Protein Level with ELISA

Adipose tissue samples (≈100 mg) were homogenized in cold phosphate-buffered saline (PBS, pH 7.4) containing protease inhibitors and centrifuged at 10,000 rpm for 10 min at 4 °C. The supernatant was collected for protein quantification. Total protein concentration was determined using a bicinchoninic acid (BCA) assay, and all samples were normalized to equal protein content before ELISA measurement.

Protein levels of PPARγ, CNR1, and ESR1 were determined using commercially available rat-specific sandwich ELISA kits according to the manufacturer’s instructions [8]. The absorbance was measured at 450 nm using a microplate reader, and concentrations were calculated from standard curves.

All assays were performed in duplicate by blinded assessors, and results were expressed as mean ± SD. Instrument models and ELISA kit details are listed in Supplementary Table S1.

### 2.6. Examination of ADIPOR1 and PPARγ Gene Expression Using qPCR

Total RNA was extracted from approximately 50 mg of adipose tissue using a commercial RNA isolation kit according to the manufacturer’s instructions. RNA purity and concentration were assessed spectrophotometrically at 260/280 nm. Complementary DNA (cDNA) was synthesized from 1 µg of total RNA using a reverse transcription kit following standard protocol conditions. The primer sequences used for quantitative real-time PCR (qPCR) are listed in Table 1. Based on the metabolic and histological findings, the PM-H group demonstrated the strongest anti-obesity activity and was therefore selected for downstream molecular expression analysis.

Primer specificity was confirmed by melt-curve analysis, and amplification efficiency ranged between 90–110%.

Quantitative real-time PCR (qPCR) was performed using SYBR Green chemistry on a real-time PCR system. Specific primers for *Adipor1*, *Pparγ*, and the reference gene *Gapdh* were designed based on published rat sequences and validated for efficiency (90–110%) and specificity via melt-curve analysis.

Amplification reactions were conducted in triplicate for each sample. Relative gene expression was calculated using the 2^−ΔΔCt^ method [19], normalized to GAPDH. Negative controls (no-template and no-RT) were included to confirm assay specificity.

All qPCR analyses were performed by blinded investigators. Primer sequences, reaction conditions, and reagent details are in Table 1. Primer sequences for ADIPOR1, PPARγ, and GAPDH were designed based on rat sequences from GenBank and synthesized commercially. Each primer pair produced a single amplicon confirmed by melt-curve analysis.

Based on the metabolic, biochemical, adiposity, and histological results, the PM-H group showed the strongest anti-obesity effect among the *P. minor* extract-treated groups. Therefore, qPCR analysis was performed on samples from the NC, HFD, ORL, and PM-H groups only (*n* = 6 rats/group) to investigate the molecular response associated with the most effective extract dose.

### 2.7. Histological Examination of Adipose Tissue

Adipose tissue samples from the perirenal region were collected immediately after euthanasia and fixed in 10% neutral buffered formalin for at least 24 h. Tissues were processed routinely, embedded in paraffin, and sectioned at 5 µm thickness using a microtome. Sections were mounted on glass slides and stained with hematoxylin and eosin (H&E) for morphological evaluation.

Histological examination was performed using a binocular microscope (Olympus CX23 LED RFS1, Olympus, Japan) at the Primate Research Center, IPB University. Adipocyte morphometric analysis was performed using digital image analysis software on randomly selected microscopic fields. Adipocyte perimeter (µm), representing the cell circumference obtained from image segmentation analysis, was quantified from multiple adipocytes per section. Measurements were independently evaluated by two blinded observers, and the mean values were used for statistical analysis.

All slides were coded, and observers performing morphometric analysis were blinded to treatment groups. Detailed information on equipment and reagents used for histological processing and imaging is provided in Supplementary Table S1.

### 2.8. Statistical Analysis

All statistical analyses were performed using SPSS version 22 (IBM Corp., Armonk, NY, USA), and a *p*-value < 0.05 was considered statistically significant. All data are expressed as the mean ± standard deviation (SD). Normality of data distribution was assessed using the Shapiro–Wilk test. For normally distributed data, differences among groups were analyzed using one-way analysis of variance (ANOVA), followed by Tukey’s post hoc test for multiple comparisons. The sample size (*n* = 6 per group) was determined based on previous studies using high-fat diet–induced obesity models, where similar group sizes were sufficient to detect significant differences in metabolic and molecular parameters. No formal protocol registration was performed; however, the study design and analysis plan were defined prior to the experiment. Animals showing signs of illness or abnormal baseline body weight would have been excluded; however, no such cases occurred. All animals that completed the experimental protocol were included in the analysis. No animals, experimental units, or data points were excluded from the analysis. All animals completed the study and were included in the final statistical analyses. The primary outcome measure was body weight gain, which was used as the main indicator of anti-obesity efficacy and guided the determination of sample size. Secondary outcomes included adiposity index, serum lipid profile, glucose levels, and molecular markers (PPARγ, CNR1, ESR1, and Adipor1). Animals were monitored daily, and predefined humane endpoints were in place; however, no animals met these criteria during the study.

## 3. Results

The collected *Padina minor* was taxonomically verified by the Oceanographic Laboratory, National Research and Innovation Agency (BRIN), Ancol, Indonesia (Specimen ID: #143810), and confirmed as *P. minor* [14] Dried *P. minor* simplicia (3.2 kg) were extracted with 19 L of 70% ethanol at room temperature. The concentrated ethanolic extract yielded 320.68 g of thick extract, corresponding to a 10.02% (*w*/*w*) yield.

### 3.1. Analysis of Chemical Contents of P. minor by GCMS and LCMS

GC–MS and LC–MS/MS analyses identified 15 major constituents in the ethanolic extract of *P. minor* (Table 2). The predominant GC–MS–detected compounds were hexadecanoic acid, palmitoleic acid, octadecanoic acid, γ-sitosterol, and stigmasta-3,5-diene. LC–MS/MS analysis revealed several abundant phenolic compounds, including chlorogenic acid, quercetin, *p*-coumaric acid, and epicatechin. These constituents are widely associated with lipid-modulating, anti-inflammatory, and metabolic regulatory activities, which may contribute to the observed anti-obesity effects.

In addition, pseudosarsasapogenin was detected, a steroidal saponin structurally related to sarsasapogenin, which has been reported to exhibit antihyperglycemic activity [20]. γ-Sitosterol is a bioactive plant sterol known to modulate lipid metabolism and glucose homeostasis [21]. The identified phenolic compounds—chlorogenic acid, quercetin, *p*-coumaric acid, and epicatechin—have been extensively reported to improve insulin sensitivity, reduce lipid accumulation, and attenuate oxidative and inflammatory stress [22]. Collectively, the presence of these bioactive constituents supports the metabolic and molecular effects observed following *P. minor* treatment.

### 3.2. Body Weight, Food Intake, and Lee Index

**Figure 1:**
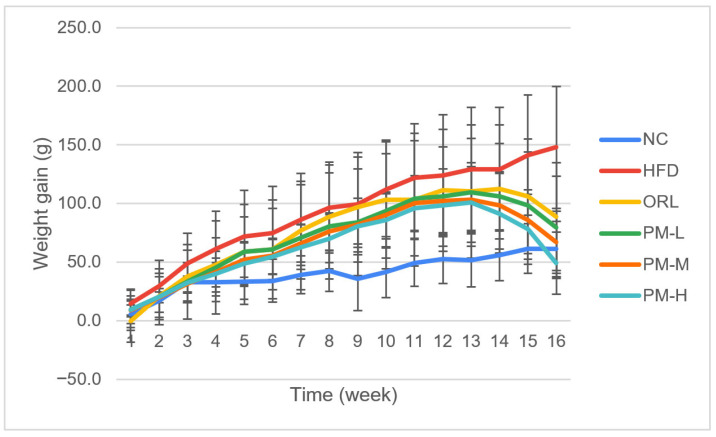
Changes in body weight during four weeks of treatment in high-fat diet–induced obese rats. Note: NC = the group that was given standard feed (non-HFD) of 58 kcal/20 g/rat, HFD = the group that was given HFD feed of 89.72 kcal/20 g/rat; ORL = the group that was given Orlistat 30 mg/kg BW; PM-L the group that was given *P. minor* extract 25 mg/kg BW; PM-M = the group that was given *P. minor* extract 50 mg/kg BW, PM-H = the group that was given *P. minor* extract 100 mg/kg BW. *n* = 6 rats/group.

No adverse events occurred, and all animals completed the study without complications. Rats fed a high-fat diet (HFD) showed a significant increase in body weight compared with the normal control (NC) group throughout the 4-week treatment period (*p* < 0.05). Administration of *P. minor* extract significantly attenuated HFD-induced weight gain in a dose-dependent manner as shown in Figure 1.

During the first week, HFD rats gained 14.8 ± 1.5 g, which was significantly higher than NC rats (5.6 ± 1.1 g; *p* < 0.05). All *P. minor*–treated groups exhibited reduced weight gain, with the greatest effect observed in the high-dose group (PM-H: 6.4 ± 1.8 g; *p* < 0.05 vs. HFD). By week 3, cumulative weight gain in HFD rats reached 49.0 ± 10.8 g, whereas PM-H and PM-M groups showed significantly lower gains (35.3 ± 7.8 g and 31.0 ± 6.8 g, respectively), comparable to the orlistat group (37.9 ± 8.4 g).

The effects of a high-fat diet (HFD) and subsequent treatments on food intake, Lee index, and metabolic parameters are presented in Table 3.

Food intake remained relatively consistent across all groups before and after HFD induction. However, following treatment, a noticeable reduction in food intake was observed in the ORL and PM-treated groups, particularly in the PM-H group (12.43 ± 0.7), compared to the HFD group (16.4 ± 1.5), suggesting a potential appetite-suppressing effect of the treatments.

The Lee index, an indicator of obesity, increased markedly after HFD induction in all experimental groups compared to the NC group, confirming successful obesity development. After treatment, the Lee index remained elevated in the HFD group (0.36 ± 0.002), whereas it was reduced in the ORL (0.31 ± 0.006) and PM-treated groups, with PM-M and PM-H showing values (0.29 ± 0.006) comparable to the NC group, indicating an improvement in adiposity.

Total cholesterol levels increased substantially following HFD administration, with the HFD group showing the highest levels (61.20 ± 2.32). After treatment, cholesterol levels remained elevated in the HFD group (65.88 ± 5.50) but were significantly reduced in the ORL and PM-treated groups. Notably, the PM-M (45.25 ± 4.37) and PM-H (43.92 ± 14.01) groups exhibited values close to those of the NC group, indicating effective lipid-lowering activity.

HDL-cholesterol levels decreased markedly after HFD induction in all groups except NC, reflecting impaired lipid metabolism. Treatment resulted in a recovery of HDL levels, particularly in the PM-M (52.32 ± 6.55) and PM-H (55.72 ± 11.58) groups, which approached or exceeded NC levels, suggesting improved lipid profile.

Triglyceride levels were significantly elevated following HFD, especially in the HFD group (217.11 ± 33.19). After treatment, triglyceride levels further increased in the HFD group (249.50 ± 39.83), while substantial reductions were observed in the ORL and PM-treated groups. The PM-H group showed the greatest improvement (105.63 ± 7.41), approaching normal levels.

Blood glucose levels showed a moderate increase after HFD induction. Post-treatment, glucose levels remained elevated in the HFD group (102.33 ± 13.19), whereas reductions were observed in all treatment groups. The ORL (85.50 ± 6.53) and PM-treated groups demonstrated glucose levels comparable to or lower than the NC group, indicating improved glycemic control.

The biochemical parameters observed in the NC group were within the normal physiological ranges for Wistar rats, confirming the validity of the experimental model.

Overall, the results demonstrate that PM treatment, particularly at higher doses, effectively ameliorates HFD-induced obesity and metabolic disturbances, as evidenced by improvements in Lee index, lipid profile, and glucose levels.

### 3.3. Effect of P. minor Extract on Visceral Fat Weight and Adiposity Index

Visceral fat mass and adiposity index were significantly increased in high-fat diet (HFD)–fed rats (21.33 ± 5.92 g and 5.84 ± 0.90%, respectively) compared with normal controls (Table 4). Treatment with *Padina minor* extract significantly reduced both parameters in a dose-dependent manner, with the strongest effect observed at 100 mg/kg BW (10.92 ± 0.95 g and 5.50 ± 0.98%), comparable to the orlistat-treated group (*p* < 0.05 vs. HFD).

Notably, the highest dose of *P. minor* extract (100 mg/kg BW) resulted in substantial improvements compared with the HFD group, including a ~67% reduction in body weight gain, ~49% reduction in visceral fat weight, ~58% reduction in triglyceride levels, ~33% reduction in total cholesterol, and ~14% reduction in blood glucose. These effects were comparable to those observed in the orlistat-treated group.

**Figure 2:**
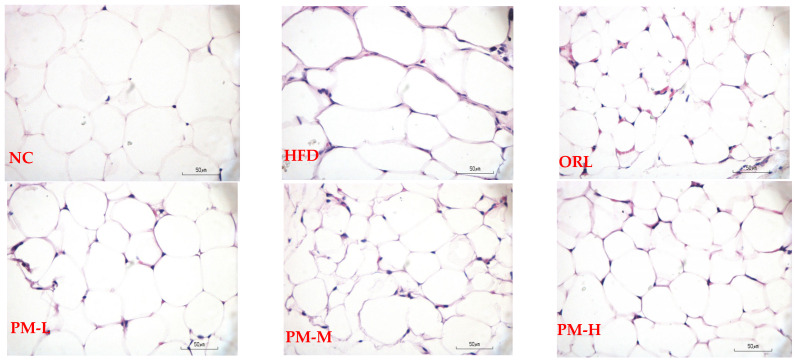
Representative histological sections of adipose tissue showing adipocyte morphology after treatment (H&E staining). Images were obtained using a binocular microscope (Olympus CX23 LED RFS1, Olympus, Japan). Note: NC = the group that was given standard feed (non-HFD) of 58 kcal/20 g/rat, HFD = the group that was given HFD feed of 89.72 kcal/20 g/rat; ORL = the group that was given Orlistat 30 mg/kg BW; PM-L the group that was given *P. minor* extract 25 mg/kg BW; PM-M = the group that was given *P. minor* extract 50 mg/kg BW, PM-H = the group that was given *P. minor* extract 100 mg/kg BW.

Histological analysis of adipose tissue revealed marked adipocyte hypertrophy in HFD rats, as indicated by increased adipocyte perimeter (349.7 ± 5.0 µm; Figure 2). Administration of *P. minor* extract significantly reduced adipocyte size, with a mean perimeter of 213.9 ± 41.5 µm in treated rats (*p* < 0.001 vs. HFD), consistent with reduced fat accumulation. Similar reductions were observed in the orlistat group.

Overall, *P. minor* extract effectively decreased visceral fat deposition, adiposity index, and adipocyte hypertrophy in HFD-induced obese rats, indicating an improvement in adipose tissue morphology and fat accumulation.

### 3.4. The Effect of P. minor Extract on ESR1, CNR, and PPAR-γ Protein Levels

ELISA analysis demonstrated significant alterations in adipose tissue protein expression in high-fat diet (HFD)–fed rats (Table 5). Compared with the normal control (NC) group, HFD rats showed a marked upregulation of PPARγ and CNR1 and a pronounced downregulation of ESR1 (*p* < 0.05), indicating obesity-associated receptor dysregulation.

Treatment with *Padina minor* extract significantly reversed these changes in a dose-dependent manner. PPARγ protein expression increased from 0.50 ± 0.208 in NC rats to 3.58 ± 0.624 in HFD rats but was significantly reduced following *P. minor* administration to 1.31 ± 0.311, 1.01 ± 0.311, and 0.88 ± 0.208 in the PM-L, PM-M, and PM-H groups, respectively (*p* < 0.05 vs. HFD), approaching levels observed in the orlistat-treated group.

Similarly, ESR1 expression was markedly reduced in HFD rats (0.21 ± 0.037) compared with NC rats (2.13 ± 0.097; *p* < 0.05). *P. minor* treatment significantly restored ESR1 protein levels in a dose-dependent manner, with the highest dose producing values close to normal (*p* < 0.05 vs. HFD).

CNR1 protein expression was strongly elevated in HFD rats (363.33 ± 47.14) but was significantly suppressed following *P. minor* treatment, with the greatest reduction observed in the PM-H group (*p* < 0.05 vs. HFD), comparable to orlistat.

Overall, *P. minor* ethanolic extract significantly modulated key adipogenic and metabolic regulators by decreasing PPARγ and CNR1 expression and restoring ESR1 levels in adipose tissue of HFD-induced obese rats.

### 3.5. Analysis of the Expression Levels of Pparγ and Adipor1 in Rat Adipose Tissue of Obesity Model Rat

qPCR analysis confirmed the protein expression findings (Table 6). In high-fat diet (HFD)–fed rats, *Padina minor* treatment significantly upregulated *Adipor1* gene expression by approximately 2.8-fold compared with the HFD group (*p* < 0.001). In contrast, Pparγ mRNA expression in the PM-H group remained lower than in the HFD group, indicating partial suppression of adipogenic signaling of HFD levels (*p* < 0.001).

These results demonstrate that *P. minor* extract modulates key genes involved in adipose tissue metabolism by enhancing *Adipor1* expression and suppressing *Pparγ* expression in obese rats.

## 4. Discussion

This study demonstrates that *Padina minor* ethanolic extract improves metabolic parameters and adipose tissue characteristics in high-fat diet (HFD)-induced obese rats. The observed reductions in body weight gain, adiposity indices, and visceral fat accumulation, together with improvements in lipid and glucose profiles, indicate that *P. minor* exerts beneficial effects on obesity-associated metabolic dysfunction. These findings are consistent with previous studies reporting the anti-obesity potential of marine-derived bioactive compounds, particularly brown algae rich in polyphenols and flavonoids [23,24].

The metabolic improvements observed in this study are in agreement with earlier reports on algal-derived compounds such as fucoxanthin and polyphenols, which have been shown to reduce adiposity and improve lipid metabolism in experimental models [25,26]. For instance, fucoxanthin has been reported to enhance lipid oxidation and reduce fat accumulation through modulation of metabolic pathways, including PPAR signaling [27,28,29,30]. Similarly, studies have demonstrated that flavonoid-rich extracts can attenuate diet-induced obesity by improving insulin sensitivity and lipid profiles [31]. Interestingly, the dose–response relationship was not strictly linear across all measured parameters. Although the PM-H group generally showed the strongest overall metabolic and molecular improvements, certain outcomes, such as adiposity index, were not proportionally lower than those observed in the PM-M group. This finding may indicate a plateau or saturation effect, which is commonly reported in phytochemical-based interventions where maximal biological activity may occur within an optimal dose range rather than increasing continuously with higher doses. Additionally, biological variability and tissue-specific responsiveness may contribute to these differences. Further dose-optimization and pharmacodynamic studies are needed to better define the therapeutic window of *P. minor* extract.

Importantly, the reduction in body weight gain in the present study was accompanied by decreased food intake, particularly at higher doses of *P. minor*. This observation is consistent with findings from previous studies indicating that modulation of cannabinoid receptor signaling (CNR1) can influence appetite regulation and energy intake [31,32,33]. Therefore, the anti-obesity effects observed here may result from a combination of reduced caloric intake and metabolic modulation, rather than a single mechanism.

At the molecular level, *P. minor* treatment was associated with downregulation of PPARγ and CNR1 and upregulation of ADIPOR1 and ESR1. These findings are broadly consistent with previous studies investigating the metabolic effects of plant- and algae-derived flavonoids, quercetin has been shown to suppress PPARγ expression and inhibit adipocyte differentiation [34,35,36], while enhancing adiponectin receptor signaling, leading to improved lipid metabolism and insulin sensitivity [37,38]. Likewise, chlorogenic acid has been reported to reduce lipid accumulation and improve glucose homeostasis through modulation of similar pathways [39,40,41]. The upregulation of *Adipor1* observed in this study aligns with clinical and experimental findings showing that increased adiponectin signaling enhances fatty acid oxidation and improves metabolic homeostasis [42,43,44].

The restoration of ESR1 expression further supports a role for estrogen-related signaling in mediating the metabolic effects of *P. minor*. Previous studies have highlighted the importance of ESR1 in regulating adipose tissue distribution and lipid metabolism, with reduced ESR1 activity being associated with obesity and metabolic dysfunction [45,46]. Therefore, the observed increase in ESR1 expression may contribute to improved adipose tissue function in treated animals.

Collectively, these findings suggest that *P. minor* extract is associated with modulation of multiple interconnected pathways involved in adipogenesis, lipid metabolism, and energy balance. This multi-target profile is consistent with the complex phytochemical composition of brown algae, where flavonoids, phenolic acids, sterols, and saponins may act synergistically to produce metabolic benefits. Similar multi-target effects have been reported in other natural product studies, supporting the concept that combinations of bioactive compounds may be more effective than single agents in addressing complex metabolic disorders such as obesity [4].

Despite these promising results, several limitations should be acknowledged. First, the study was conducted in an animal model, and translation to human physiology remains uncertain. Second, the relatively small sample size may limit statistical robustness. Third, although associations with key molecular markers were identified, causal relationships between these pathways and the observed metabolic outcomes were not directly established. Future studies incorporating pathway-specific inhibition, gene knockdown approaches, or metabolomic analyses would provide stronger mechanistic evidence. Additionally, long-term safety and toxicity evaluations are necessary before considering clinical application.

Although the extract contained multiple bioactive constituents, including flavonoids, phenolic acids, sterols, and saponins, the present study did not determine the relative contribution of individual compounds to the observed anti-obesity effects. Because the study focused on evaluation of the crude extract, compound-specific activity and quantitative correlation analyses were beyond the scope of the current work. Future studies employing bioactivity-guided fractionation, metabolite correlation analysis, and isolation of active constituents are warranted to clarify which compounds contribute most strongly to the observed metabolic effects.

In summary, the present findings are consistent with and extend previous research on marine-derived bioactive compounds, demonstrating that Padina minor ethanolic extract improves metabolic parameters and adipose tissue function in HFD-induced obese rats. These effects are likely associated with modulation of multiple signaling pathways, including PPARγ, ADIPOR1, CNR1, and ESR1. While further mechanistic and clinical studies are required, this study supports the potential of *P. minor* as a promising marine-derived nutraceutical candidate for obesity management.

Although no overt adverse effects were observed during the study period, comprehensive toxicological evaluation, including assessment of non-adipose organ histology and organ weights, was beyond the scope of the present study and should be addressed in future investigations.

A limitation of the present study is that formal stability testing of individual bioactive compounds during storage was not performed. Although the extract was stored under low-temperature, light-protected conditions designed to minimize degradation, the stability of polyphenols and other sensitive constituents over the storage period remains to be further investigated.

## 5. Conclusions

This study demonstrates that Padina minor ethanolic extract effectively counteracts high-fat diet–induced obesity through coordinated modulation of adipose tissue metabolism and systemic metabolic parameters. Treatment with *P. minor* significantly reduced body weight gain, adiposity indices, visceral fat accumulation, dyslipidemia, and hyperglycemia in obese rats.

At the molecular level, *P. minor* suppresses adipogenic signaling by downregulating PPARγ and CNR1 while enhancing lipid metabolic pathways through upregulation of *Adipor1* and ESR1. These coordinated molecular changes shift adipose tissue function from lipid storage toward increased lipid utilization, consistent with the observed improvements in adipocyte morphology and metabolic homeostasis.

Collectively, these findings indicate that Padina minor exerts anti-obesity effects by inhibiting adipogenesis and promoting lipid metabolism through multi-target regulation of key metabolic pathways. The presence of bioactive compounds such as flavonoids, phenolic acids, sterols, and saponins likely contributes to these synergistic effects.

Overall, Padina minor represents a promising marine-derived natural product with potential application as a complementary therapeutic strategy for obesity management. Further studies are warranted to elucidate its long-term safety, isolate active constituents, and evaluate clinical efficacy.

## Figures and Tables

**Table 1:** Primer sequences used for qPCR analysis.

Gene	Forward Primer (5′–3′)	Reverse Primer (5′–3′)	Product Size (bp)
*Adipor1*	CAGCGGTTCTGGAAGGAGAT	TGCTGTTGTTGCTGATGAGG	162
*Pparγ*	CCTGTTGACCCAGAGCATGA	GGAATGCGAGTGGTCTTCCA	184
*Gapdh*	TGTGTCCGTCGTGGATCTGA	TTGCTGTTGAAGTCGCAGGAG	143

**Table 2:** Phytochemical constituents of *Padina minor* ethanolic extract identified by GC–MS and LC–MS/MS.

Identification		
GC-MS		LC–MS/MS
Tetradecanoic acid	10E, 12Z-Octadecadienoic acid	Chlorogenic acid
Hexadecanoic acid	(9E, 11E)-Octadecadienoic acid	Quercetin
Palmitoleic acid	Pseudosarsasapogenin	p-Coumaric acid
n-Hexadecanoic acid	2-Methyltriacontane	Epicatechin
9-Octadecanoic acid (Z)—Methyl ester	Stigmasta-3,5-diene	
9-Octadecanoic acid, (E)-	gamma-sitosterol	
Octadecanoic acid		

**Table 3:** Effects of high-fat diet and *Padina minor* treatment on food intake, Lee index, and metabolic parameters in experimental groups.

Parameter	Time Point	NC	HFD	ORL	PM-L	PM-M	PM-H
Food intake (g/day)	Before HFD	16.00 ± 2.40	15.80 ± 2.30	15.80 ± 2.30	15.80 ± 2.30	15.80 ± 2.30	15.80 ± 2.30
	After HFD	15.88 ± 2.30	16.00 ± 2.40	16.00 ± 2.40	16.00 ± 2.40	16.00 ± 2.40	16.00 ± 2.40
	After treatment	16.10 ± 1.60	16.40 ± 1.50	14.30 ± 0.90	13.40 ± 0.50	13.10 ± 0.90	12.43 ± 0.70
Lee index	Before HFD	0.28 ± 0.002	0.29 ± 0.002	0.29 ± 0.002	0.29 ± 0.002	0.29 ± 0.002	0.29 ± 0.002
	After HFD	0.29 ± 0.001	0.33 ± 0.003 ***	0.33 ± 0.003 ***	0.33 ± 0.003 ***	0.33 ± 0.003 ***	0.33 ± 0.003 ***
	After treatment	0.29 ± 0.002	0.36 ± 0.002 ***	0.31 ± 0.006 ##	0.30 ± 0.006 ###	0.29 ± 0.006 ###	0.29 ± 0.006 ###
Total cholesterol (mg/dL)	Before HFD	41.79 ± 10.79	44.24 ± 5.94	40.49 ± 6.33	39.48 ± 2.85	40.58 ± 4.37	41.31 ± 0.88
	After HFD	45.36 ± 8.13	61.20 ± 2.32 ***	58.06 ± 13.88 ***	57.29 ± 5.13 ***	57.36 ± 11.77 ***	60.42 ± 2.95 ***
	After treatment	44.36 ± 7.27	65.88 ± 5.50 ***	42.44 ± 4.19 ###	53.81 ± 0.91 #	45.25 ± 4.37 ###	43.92 ± 14.01 ###
HDL-cholesterol (mg/dL)	Before HFD	53.24 ± 5.37	54.51 ± 13.05	53.24 ± 8.95	54.69 ± 9.52	54.12 ± 12.45	54.30 ± 20.32
	After HFD	55.83 ± 9.57	34.56 ± 5.35 ***	34.52 ± 8.18 ***	32.36 ± 9.19 ***	33.75 ± 5.08 ***	31.98 ± 9.98 ***
	After treatment	51.11 ± 6.72	39.83 ± 5.67 ***	50.45 ± 9.36 ##	47.53 ± 7.43 #	52.32 ± 6.55 ###	55.72 ± 11.58 ###
Triglyceride (mg/dL)	Before HFD	129.49 ± 84.57	125.09 ± 35.96	115.60 ± 3.36	122.75 ± 2.36	125.68 ± 41.10	117.05 ± 45.30
	After HFD	109.25 ± 60.28	217.11 ± 33.19 ***	212.88 ± 36.36 ***	221.35 ± 5.16 ***	222.22 ± 24.31 ***	213.50 ± 9.09 ***
	After treatment	101.95 ± 28.75	249.50 ± 39.83 ***	129.17 ± 41.66 ###	137.23 ± 13.42 ##	125.40 ± 9.35 ###	105.63 ± 7.41 ###
Glucose (mg/dL)	Before HFD	88.33 ± 5.68	88.25 ± 12.12	88.67 ± 5.43	86.33 ± 5.43	87.17 ± 9.30	87.50 ± 6.60
	After HFD	87.83 ± 6.05	96.00 ± 6.06 *	98.75 ± 6.08 **	91.67 ± 0.58 *	94.00 ± 3.61 *	94.00 ± 1.00 *
	After treatment	81.00 ± 6.40	102.33 ± 13.19 ***	85.50 ± 6.53 ##	87.17 ± 6.24 #	87.33 ± 2.88 #	88.17 ± 1.94 #

Note: Data are expressed as mean ± SD (*n* = 6 rats/group). NC = normal control group fed standard diet (58 kcal/20 g/rat); HFD = high-fat diet group (89.72 kcal/20 g/rat); ORL = orlistat-treated group (30 mg/kg BW); PM-L = *P. minor* extract 25 mg/kg BW; PM-M = *P. minor* extract 50 mg/kg BW; PM-H = *P. minor* extract 100 mg/kg BW. Statistical significance was determined using one-way ANOVA followed by Tukey’s post hoc test. * *p* < 0.05, ** *p* < 0.01, *** *p* < 0.001 versus NC group; # *p* < 0.05, ## *p* < 0.01, ### *p* < 0.001 versus HFD group. Reference ranges for healthy Wistar rats are as follows: total cholesterol (40–80 mg/dL), HDL-cholesterol (25–60 mg/dL), triglycerides (50–150 mg/dL), and fasting glucose (70–120 mg/dL). Values may vary depending on age, diet, and laboratory conditions.

**Table 4:** Effects of *Padina minor* extract on visceral fat weight, adiposity index, and adipocyte perimeter in obese rats.

Parameter	NC	HFD	ORL	PM-L	PM-M	PM-H
Visceral fat weight (g)	6.22 ± 3.15	21.33 ± 5.92 ***	13.57 ± 2.47 ##	12.95 ± 1.52 ** ##	11.42 ± 1.06 *** ###	10.92 ± 0.95 *** ###
Adiposity index (%)	2.11 ± 0.84	5.84 ± 0.90 ***	5.09 ± 0.44 ##	4.71 ± 0.62 * ##	5.05 ± 0.44 ##	5.50 ± 0.98 #
Adipocyte perimeter (µm)	223.05 ± 10.82	349.71 ± 5.01 ***	199.89 ± 51.96 ###	173.59 ± 44.74 ###	194.07 ± 15.06 ###	213.91 ± 41.53 ##

Note: Data are expressed as mean ± SD (*n* = 6 rats/group). NC = normal control group fed standard diet (58 kcal/20 g/rat); HFD = high-fat diet group (89.72 kcal/20 g/rat); ORL = orlistat-treated group (30 mg/kg BW); PM-L = *P. minor* extract 25 mg/kg BW; PM-M = *P. minor* extract 50 mg/kg BW; PM-H = *P. minor* extract 100 mg/kg BW. Statistical significance was determined using one-way ANOVA followed by Tukey’s post hoc test. * *p* < 0.05, ** *p* < 0.01, *** *p* < 0.001 versus NC group; # *p* < 0.05, ## *p* < 0.01, ### *p* < 0.001 versus HFD group.

**Table 5:** Relative expression levels of ESR1, PPARγ, and CNR1 in adipose tissue of obese rats.

Group	ESR1	PPARγ	CNR1 (pg/mL)
NC	2.13 ± 0.10	0.50 ± 0.21	21.67 ± 11.79
HFD	0.21 ± 0.04 ***	3.58 ± 0.62 ***	363.33 ± 47.14 ***
ORL	1.57 ± 0.05 *** ###	1.68 ± 0.21 ** ##	246.67 ± 33.33 ***
PM-L	0.64 ± 0.17 ** ##	1.31 ± 0.31 * ##	BDL
PM-M	1.37 ± 0.26 *** ###	1.01 ± 0.31 #	BDL
PM-H	2.10 ± 0.80 ###	0.94 ± 0.67 ##	121.67 ± 11.79 ** ###

Note: Data are expressed as mean ± SD (*n* = 6 rats/group). NC = normal control group fed standard diet (58 kcal/20 g/rat); HFD = high-fat diet group (89.72 kcal/20 g/rat); ORL = orlistat-treated group (30 mg/kg BW); PM-L = *P. minor* extract 25 mg/kg BW; PM-M = *P. minor* extract 50 mg/kg BW; PM-H = *P. minor* extract 100 mg/kg BW. Statistical significance was determined using one-way ANOVA followed by Tukey’s post hoc test. * *p* < 0.05, ** *p* < 0.01, *** *p* < 0.001 versus NC group; # *p* < 0.05, ## *p* < 0.01, ### *p* < 0.001 versus HFD group. BDL = below quantifiable range.

**Table 6:** Expression levels of *Pparγ* and *Adipor1* in adipose tissue of obese rats.

Genes	NC	HFD	ORL	PM-H
*Adipor1*	1.00 ± 0.00	3.27 ± 0.83 ***	2.09 ± 0.73 **	1.92 ± 0.50 ** ##
*Pparγ*	1.00 ± 0.00	2.82 ± 1.61 **	0.98 ± 0.18	1.98 ± 0.68 *

Data are expressed as mean ± SD (*n* = 6 rats/group). NC = normal control group fed standard diet (58 kcal/20 g/rat); HFD = high-fat diet group (89.72 kcal/20 g/rat); ORL = orlistat-treated group (30 mg/kg BW); PM-L = *P. minor* extract 25 mg/kg BW; PM-M = *P. minor* extract 50 mg/kg BW; PM-H = *P. minor* extract 100 mg/kg BW. Statistical significance was determined using one-way ANOVA followed by Tukey’s post hoc test. * *p* < 0.05, ** *p* < 0.01, *** *p* < 0.001 versus NC group; ## *p* < 0.01. PM-L and PM-M groups were not included in qPCR analysis because the assay was focused on the PM-H group, which showed the strongest overall anti-obesity effect.

## Data Availability

The datasets generated during the current study are available from the corresponding author on reasonable request.

## Supplementary Materials

The following supporting information can be downloaded at: https://www.mdpi.com/article/10.3390/nu18101572/s1, Table S1: Instruments and reagents used in the study, including analytical equipment, biochemical assay kits, molecular biology reagents, and histological materials. Table S2: Major compounds identified in the 70% ethanolic extract of *Padina minor* by GC–FID, including retention time, retention index, chemical class, and relative abundance (%TIC). Table S3: Tentatively identified compounds in the 70% ethanolic extract of *P. minor* by LC–MS/MS, including molecular formula, fragment ions, database matches, and compound classification. Table S4: Primer sequences used for quantitative real-time PCR (qPCR), including forward and reverse primers and product sizes.
