# Supplementary material for: Marine-Derived Padina Minor Extract Improves Lipid and Glucose Metabolism in Obese Rats: Evidence for PPARγ and ADIPOR1 Modulation
Source: Nutrients. 2026 May 15;18(10):1572. doi: 10.3390/nu18101572 (PMC13209200; doi:10.3390/nu18101572)
Supplement: Supplementary file 1 [file nutrients-18-01572-s001.zip › nutrients-4286952-supplementary.pdf]

Table S1. Instruments and reagents used in this study

Category	Equipment / Reagent	Model / Kit	Manufacturer	Country
Analytical instruments	Analytical balance	PAJ 1003	Ohaus Corporation	USA
	Biochemistry analyzer	BS-120	Mindray Bio-Medical Electronics Co., Ltd.	China
	UV–Vis spectrophotometer	NanoDrop 2000	Thermo Fisher Scientific	USA
	Centrifuge	EBA 20	Andreas Hettich GmbH & Co.	Germany
	PCR system	CFX96	Bio-Rad Laboratories	USA
	Microplate reader	Epoch 2	BioTek Instruments	USA
Biochemical assay kits	Triglyceride assay kit	Triglyceride GPO–PAP	DiaSys Diagnostic Systems GmbH	Germany
	Total cholesterol assay kit	CHOD–PAP	DiaSys Diagnostic Systems GmbH	Germany
	HDL assay kit	HDL–C plus	DiaSys Diagnostic Systems GmbH	Germany
	Glucose assay kit	GOD–PAP	DiaSys Diagnostic Systems GmbH	Germany
Molecular analysis	RNA extraction kit	PureLink RNA Mini Kit	Thermo Fisher Scientific	USA
	cDNA synthesis kit	ReverTra Ace	Toyobo Co., Ltd.	Japan

		qPCR RT Kit		
	qPCR reagents	SYBR Green Master Mix	Bio-Rad Laboratories	USA
Histology	Tissue fixative	10% neutral buffered formalin	Merck	Germany
	Embedding and staining reagents	Paraffin, hematoxylin–eosin	Sigma-Aldrich	USA

Supplementary Table S2. Major compounds identified in the 70% ethanolic extract of *Padina minor* by GC–FID.

No.	RT (min)	Tentative compound	Retention index (RI)	Major ions (m/z)*	Reference / Library match	Chemical class	Relative content (% TIC)	Identification method
1	8.32	Tetradecanoic acid (myristic acid)	1689	74, 87, 143	NIST / standard RT	Fatty acid	5.2	RT + standard
2	10.47	Pentadecanoic acid	1796	74, 87, 143	NIST	Fatty acid	3.7	RT + library
3	12.65	Hexadecanoic acid (palmitic acid)	1958	74, 87, 143	Standard / NIST	Fatty acid	15.8	RT + standard
4	14.18	9-Octadecenoic acid (oleic acid)	2142	55, 69, 83	NIST	Fatty acid	18.3	RT + library
5	14.86	Octadecanoic acid (stearic acid)	2177	74, 87, 143	Standard / NIST	Fatty acid	9.6	RT + standard
6	16.45	Phytol	2179	67, 71, 123	NIST	Diterpene alcohol	7.1	RT + library
7	18.74	γ-Sitosterol	3122	129, 255, 396	NIST	Sterol	6.8	RT + library
8	19.52	Stigmasterol	3108	129, 255, 394	NIST	Sterol	5.4	RT + library
9	21.11	Squalene	2825	69, 81, 95	NIST	Triterpene	4.5	RT + library
10	22.48	Eicosanoic acid (arachidic acid)	2301	74, 87, 143	NIST	Fatty acid	3.2	RT + library

* For FID analyses, major ions refer to reference spectra from NIST or confirmation GC–MS runs.

Notes: Identification was performed by comparing retention times and retention indices with those of authentic standards and NIST library references. Quantification was expressed as the relative percentage of total ion current (% TIC). Fatty acids and sterols represented the predominant chemical classes in the 70% ethanolic extract of *Padina minor*.

Supplementary Table S3. Tentatively identified compounds in the 70% ethanolic extract of Padina minor by LC–MS/MS

No.	RT (min)	[M–H]⁻ / [M+H]⁺ (m/z)	Molecular formula	Tentative identification	MS/MS fragment ions (m/z)	Error (ppm)	Database / Reference	Class	Relative abundance (%TIC)
1	2.15	169.014	$C_7H_6O_5$	Gallic acid	125, 97, 79	3.1	METLIN / HMDB	Phenolic acid	2.8
2	3.42	289.072	$C_{15}H_{14}O_6$	Catechin	245, 205, 179	2.6	METLIN / mzCloud	Flavonoid	4.2
3	5.27	371.061	$C_{18}H_{12}O_9$	Phloroglucinol trimer	353, 249, 167	4.4	MassBank	Phlorotannin	3.7
4	7.84	255.233	$C_{16}H_{32}O_2$	Palmitic acid	255, 237, 213	1.9	METLIN	Fatty acid	8.5
5	9.12	283.263	$C_{18}H_{36}O_2$	Stearic acid	255, 283, 129	2.8	HMDB	Fatty acid	6.3
6	10.45	301.035	$C_{15}H_{10}O_7$	Quercetin	179, 151, 121	3.3	METLIN / mzCloud	Flavonoid	3.9
7	11.03	457.378	$C_{29}H_{46}O_4$	Stigmasterol derivative	255, 281, 129	2.4	METLIN	Sterol	5.6
8	12.78	621.074	$C_{30}H_{22}O_{15}$	Eckol (phlorotannin)	497, 373, 249	4.7	MassBank	Phlorotannin	4.8
9	14.63	465.102	$C_{21}H_{20}O_{12}$	Rutin	303, 271,	3.2	mzCloud	Flavonoid glycoside	3.1

10	16.45	595.153	C27H30O15	Luteolin-7-O-rutinoside	285, 151, 133	4.1	METLIN	Flavonoid glycoside	2.9

Notes: Tentative identification was based on accurate mass (±5 ppm), MS/MS fragmentation pattern, and comparison with databases (METLIN, MassBank, mzCloud, HMDB). Relative abundance is expressed as % of total ion current (TIC). Phlorotannins, flavonoids, fatty acids, and sterols represent the main chemical classes detected in the 70% ethanolic extract of Padina minor.

Table S4. Primer Sequences Used for Quantitative Real-Time PCR (qPCR)

Gene	Forward Primer (5′–3′)	Reverse Primer (5′–3′)	Product Size (bp)
Adipor1	CAGCGGTTCTGGAAGGAGAT	TGCTGTTGTTGCTGATGAGG	162
Pparγ	CCTGTTGACCCAGAGCATGA	GGAATGCGAGTGGTCTTCCA	184
Gapdh	TGTGTCCGTCGTGGATCTGA	TTGCTGTTGAAGTCGCAGGAG	143

All primer sequences were designed based on Rattus norvegicus gene sequences from GenBank. Primer efficiency (90–110%) and specificity were validated by melt-curve analysis.
